# Dangguijakyak-san ameliorates memory deficits in ovariectomized mice by upregulating hippocampal estrogen synthesis

**DOI:** 10.1186/s12906-017-2015-6

**Published:** 2017-11-25

**Authors:** Deok-Sang Hwang, Namkwon Kim, Jin Gyu Choi, Hyo Geun Kim, Hocheol Kim, Myung Sook Oh

**Affiliations:** 1 Department of Oriental Gynecology, College of Oriental Medicine, Kyung Hee University, 26, Kyungheedae-ro, Dongdaemun-gu, Seoul, 02447 Republic of Korea; 2 Department of Life and Nanopharmaceutical Sciences, Graduate School, Kyung Hee University, 26, Kyungheedae-ro, Dongdaemun-gu, Seoul, 02447 Republic of Korea; 3 Department of Oriental Pharmaceutical Science, College of Pharmacy and Kyung Hee East-West Pharmaceutical Research Institute, Kyung Hee University, 26, Kyungheedae-ro, Dongdaemun-gu, Seoul, 02447 Republic of Korea; 4 Department of Herbal Pharmacology, College of Korean Medicine, Kyung Hee University, 26, Kyungheedae-ro, Dongdaemun-gu, Seoul, 02447 Republic of Korea

**Keywords:** Dangguijakyak-san, Hippocampal estrogen, Memory, Ovariectomy

## Abstract

**Background:**

Dangguijakyak-san (DJS) is an herbal formulation that has been clinically applicable for treating postmenopausal symptoms and neurological disorders. It is reported that hippocampal estrogen attenuates memory impairment via neuroprotection and synaptogenesis. However, the effect of DJS on hippocampal estrogen synthesis remains unknown. In this study, we explored the effect of DJS and its neuroprotective mechanism against memory impairment in ovariectomized (OVX) mice, with respect to hippocampal estrogen stimulation.

**Methods:**

Cell cultures were prepared from the hippocampi of 18-day-old embryos from timed pregnant Sprague–Dawley rats. The hippocampi were dissected, collected, dissociated, and plated in 60-mm dishes. The cells were treated with DJS for 48 h and the supernatant was collected to determine estrogen levels. Female ICR mice (8-weeks-old) were housed for 1 week and ovariectomy was performed to remove the influence of ovary-synthesized estrogens. Following a 2-week post-surgical recovery period, the mice were administrated with DJS (50 and 100 mg/kg/day, *p.o.*) or 17β-estradiol (200 μg/kg/day, *i.p.*) once daily for 21 days. Hippocampal and serum estrogen levels were determined using enzyme-linked immunosorbent assay kit. Memory behavioral tests, western blot, and immunohistochemical analyses were performed to evaluate the neuroprotective effects of DJS in this model.

**Results:**

DJS treatment promoted estrogen synthesis in primary hippocampal cells and the hippocampus of OVX mice, resulting in the amelioration of OVX-induced memory impairment. Hippocampal estrogen stimulated by DJS treatment contributed to the activation of cAMP response element-binding protein and synaptic protein in OVX mice.

**Conclusion:**

DJS may attenuate memory deficits in postmenopausal women via hippocampal estrogen synthesis.

**Electronic supplementary material:**

The online version of this article (10.1186/s12906-017-2015-6) contains supplementary material, which is available to authorized users.

## Background

It is well known that estrogen plays an indispensable role in cognitive function [1]. Estrogen regulates the dendritic spine density and length in various regions of the brain and enhances long-term potentiation (LTP) at the CA3–CA1 synapse [2–5] via both cell nuclear and membrane receptors [6, 7]. Many studies have reported the molecular mechanisms associated with memory-enhancing effects of estrogen. Estrogen regulates the expression of essential extracellular-signal-regulated kinase (ERK)/cyclic-AMP response element-binding protein (CREB) signaling in long-term hippocampal memory in neurons [8–11] and synaptic protein, including synaptophysin (SYN) and postsynaptic density protein-95 (PSD-95) [12–14]. Generally, estrogen is regarded as being synthesized in the ovary and transported to the brain via the blood-brain-barrier [15–17]. Interestingly, it was reported that estrogen is synthesized from cholesterol and androgen precursor [18, 19] by neurons and glia in numerous brain regions including the hippocampus, hypothalamus, and cerebral cortex [20–22]. Even though the relative contributions of ovary-synthesized estrogen and brain-synthesized estrogen in memory function have not yet been fully elucidated, it is reported that brain-synthesized estrogen has an important role in memory formation by the preservation of hippocampal synapses [23–25]. Therefore, stimulating estrogen synthesis in the hippocampus will be a new strategy for attenuating memory impairment due to ovary dysfunction status at the postmenopausal stage.

Dangguijakyak-san (DJS), also called Tokishakuyakusan in Japan and Danggui-Shaoyao-san in China, is a traditional and popular herbal formula that consists of six traditional herbs and is widely used in neuro-associated disorders after menopause in women [26]. Many studies have demonstrated that DJS is involved in memory functions: DJS enhances LTP by ERK/CREB/brain-derived neurotrophic factor cascade in the hippocampus [27]. It ameliorates memory impairment and increases neurogenesis through modulating the Akt/glycogen synthase kinase-3 beta signaling in the bilateral common carotid artery occlusion-induced ischemia model [28]. Administering DJS and its fraction ameliorate cognitive dysfunction caused by amyloid beta in mice via decreasing the contents and deposition of amyloid beta [29]. Additionally, Toriizuka K et al. reported that DJS improved memory in ovariectomized (OVX) mice via regulating choline acetyltransferase and increasing norepinephrine contents in the brain regions [30]. Moreover, the administration of DJS results in an increase in plasma estrogen in the OVX rat model [31]. We supposed that memory primarily depends on hippocampal estrogen synthesis in an OVX model because it is difficult to synthesize estrogen in the ovaries. However, it is not yet clear whether DJS can synthesize estrogen in the hippocampus in an OVX model.

Thus, in this study, we explored whether the memory improvement effect of DJS is due to hippocampal estrogen synthesis and its related mechanisms focusing on synapse consolidation by assessing the expression of phosphorylated CREB (pCREB), SYN, and PSD-95 in an OVX model. We measured estrogen levels in primary hippocampal cells and the hippocampus of female mice under postmenopausal conditions after DJS treatment.

## Methods

### Materials

Neurobasal media (NM), B27, and penicillin-streptomycin (P/S) were purchased from Gibco Industries Inc. (Auckland, NZ). Rabbit monoclonal anti-pCREB and rabbit monoclonal anti-CREB were purchased from Santa Cruz Biotechnology, Inc. (Santa Cruz, CA, USA). Rabbit polyclonal anti-PSD-95 was purchased from Abcam (Cambridge, UK). Phosphate buffered saline (PBS), phosphatase inhibitor cocktail, 17β-estradiol (EST), 2-methyl-2-butanol, 2,2,2-tribromoethanol and mouse monoclonal anti-SYN were purchased from Sigma-Aldrich (St. Louis, MO, USA). Anti-rabbit and mouse-horse radish peroxidase secondary antibodies were purchased from Assay Designs Inc. (Ann Arbor, MI, USA). The EST high sensitivity enzyme-linked immunosorbent assay (ELISA) kit was purchased from Enzo Life Sciences Inc. (Farmingdale, NY, USA). Other reagents for Western blotting were purchased from Bio-Rad Laboratories (Hercules, CA, USA).

### Preparation of DJS

The water extract of DJS was the same as that used in our previous studies [32–34] in which it was standardized using paeoniflorin and albiflorin [32]. Briefly, we purchased individual dried herbs of DJS from Jung Do Herbal Drug Co. Ltd. (Seoul, Korea). Each herb was deposited at Department of Medicinal Herbology, College of Pharmacy, Kyung Hee University, Seoul, Korea. DJS consisted of 10 g Paeoniae Radix (*Paeonia lactiflora* Pallas, Paeoniaceae, number of voucher specimen: DBH14120301), 6 g Cnidii Rhizoma (*Cnidium officinale* Makino, Umbelliferae, number of voucher specimen: DBH16022422), 6 g Alismatis Rhizoma (*Alisma orientale* Juzepezuk, Alismataceae, number of voucher specimen: DBH16022411), 3 g Angelicae Gigantis Radix (*Angelica gigas* Nakai, Umbelliferae, number of voucher specimen: DBH16021001), 3 g Poria (*Poria cocos* Wolf, Polyporaceae, number of voucher specimen: DBH16022404), and 3 g Atractylodis Rhizoma Alba (*Atractylodes macrocephala* Koidzumi, Compositae, number of voucher specimen: DBH14111011). The mixed dried herbs were boiled with 10-fold distilled water for 2 h at 100 °C based on the way of decocting DJS in the clinical use. The suspension was filtered, lyophilized, yielding 20.16% of powder, and kept at −20 °C. This powder was dissolved in an appropriate vehicle before each experiment.

### Animals, surgery, and treatment

Female ICR mice (age, 8 weeks; weight, 32–35 g) were purchased from Daehan Biolink (Eumseong, Korea) under specific pathogen free status. The animals were randomly divided and housed 10 mice per cage, had free access to water and food, and were maintained under constant temperature (23 ± 1 °C), humidity (60 ± 10%), and a 12 h light/dark cycle. The animals were treated and cared for in accordance with the Animal Care and Use Guidelines issued by Kyung Hee University, Korea. The experimental animal protocols were approved by the Institutional Animal Care and Use Committee of Kyung Hee University, Korea (KHUASP-15-010). After 1 week of acclimation, OVX was performed to remove the influence of ovary-synthesized estrogens under anesthetics using intraperitoneally injection of 2,2,2-tribromoethaol in 2-methyl-2-butanol. After OVX surgery, there were no adverse events in mice. Following a 2-week post-surgical recovery period, the mice were divided randomly into five groups (*n* = 10/group for behavior tests to meet the statistical significance, total 50 mice): (1) the sham group (non-OVX and vehicle-treated), (2) the OVX group (OVX-lesioned and vehicle-treated group), (3) DJS 50 mg/kg/day group (OVX-lesioned and DJS 50 mg/kg/day; *p.o.*), (4) DJS 100 mg/kg/day group (OVX-lesioned and DJS 100 mg/kg/day; *p.o.*), and (5) EST group (OVX-lesioned and EST 200 μg/kg/day; *i.p.*). The DJS and EST groups were administrated once daily during 21 days and the sham and OVX groups were treated with the same volume of vehicle (Additional file 1).

### Novel object recognition test (NORT)

The NORT was carried out in a black open field box (45 × 45 × 50 cm) as described previously [35]. Results are expressed as a percentage of novel object recognition time: Novel object recognition index = [(time exploring novel object)/ (time exploring novel object + time exploring familiar object)] × 100.

### Y-maze test

The Y-maze test was carried out in a three-arm horizontal maze (each arm had a 120° angle and was 40 cm long, 3 cm wide, and 12 cm high) as described previously [36]. The number of arm entries was recorded manually for each mouse over an 8 min period. Actual alternation was defined as entry into all three arms consecutively, such as ABC, CAB, or BCA. Results are expressed as a percentage of spontaneous alternation using the following equation: % Spontaneous alternation = [(number of alternations)/ (total arm entries −2)] × 100.

### Brain tissue preparation

The mice were decapitated after the behavioral tests, and their brains were collected rapidly and excised. Hippocampal tissues were stored at −70 °C until the ELISA and western blot were performed.

### Cell culture

Cell cultures were prepared from the hippocampi of 18-day-old embryos from timed pregnant Sprague–Dawley rats (Daehan Biolink). The hippocampi were dissected, collected, dissociated, and plated in 60-mm dishes at a density of 1 × 10^6^ cells/dish. The cultures were maintained in a humidified incubator of 5% CO_2_ at 37 °C in NM with 2 mM glutamine, 2% B27, and 1% P/S. After 3 days of incubation, the medium was replaced with a new medium. The cells were treated with DJS for 48 h on day 13, and the supernatant was collected.

### Determination of EST using ELISA

The concentration of EST was detected using the ELISA kit according to the manufacturer’s instructions. Briefly, the supernatants (*n* = 5/group) from homogenized hippocampi, hippocampal neurons, and standards were incubated on a pre-coated DxS immunoglobulin G immunoplate with EST conjugate and antibody overnight at 4 °C. The plate was washed at least three times with buffer and incubated with para-nitrophenylphosphate substrate solution, which was catalyzed by alkaline phosphatase on the EST conjugate. After incubation for 1 h, the stop solution was added to each well. The plate was read at 405 nm using a spectrophotometer (Versamax microplate reader; Molecular Devices, Sunnyvale, CA, USA) and concentrations of EST were determined in the sample solution using a EST standard calibration curve. Protein from the hippocampal samples was normalized using the Bio-Rad assay for total protein determination.

### Western blotting

Homogenized hippocampal lysates (*n* = 5/group) were separated on 12% sodium dodecyl sulfatepolyacrylamide gel electrophoresis and transferred to a polyvinylidene fluoride membrane. The membranes were incubated with 5% skim milk in Tween-20 for 1 h and then with primary antibodies (1:5000 of pCREB, and CREB and 1:1000 of SYN, PSD-95, and β-actin) overnight at 4 °C, followed by incubation with horseradish peroxidase-conjugated secondary antibodies for 1 h. Immunoreactive-bands were detected using an enhanced chemiluminescence detection kit, and the LAS-4000 mini system (Fujifilm Corp., Tokyo, Japan) was used for visualization. Bands intensity was normalized to the non-phospho form band or the β-actin band using Multi Gauge software (Fujifilm Corp.).

### Statistical analysis

All statistical parameters were calculated using GraphPad Prism software (ver. 5.0; GraphPad Software Inc., San Diego, CA, USA). Values are expressed as means ± standard error of the mean. Results were analyzed by one-way analysis of variance (ANOVA) followed by Tukey’s *post-hoc* test. A *p*-value <0.05 was considered significant.

## Results

### DJS stimulates hippocampal estrogen synthesis both in the primary cells and in the mouse hippocampus

**Fig. 1:**
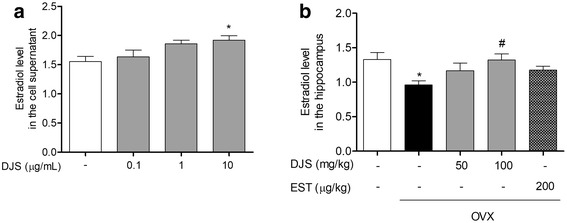
Effects of DJS on hippocampal estrogen synthesis in primary hippocampal cells and hippocampus of OVX mice. Rat primary hippocampal cells were treated with the DJS for 48 h. Female mice were administrated with DJS at the concentration of 50 and 100 mg/kg/day and EST at 200 μg/kg/day for 21 days after OVX surgery. Then, EST levels in the supernatant of cell culture (**a**) and hippocampus (**b**) were measured by ELISA. Values are means ± standard error. *p < 0.05 compared with the sham group, #*p* < 0.05 compared with the OVX group

Increasing evidence indicates that hippocampus-derived estrogen contributes to promoting synaptic plasticity and neuroprotective actions rather than estrogen synthesized from gonads [37]. We investigated whether DJS affects hippocampal estrogen synthesis in vitro and in vivo. The treatment of primary hippocampal cells with 10 μg/mL DJS for 48 h increased the levels of EST compared to the untreated group (Fig. 1A). Furthermore, to confirm the effects of DJS in vivo, we administered DJS at 50 and 100 mg/kg/day orally to mice for 21 days starting from 2 weeks after OVX. The levels of hippocampal EST in the OVX group were significantly lower than those in the sham group but were significantly increased by DJS treatment at 100 mg/kg/day (Fig. 1B).

### DJS ameliorates OVX-induced memory impairments in mice

**Fig. 2:**
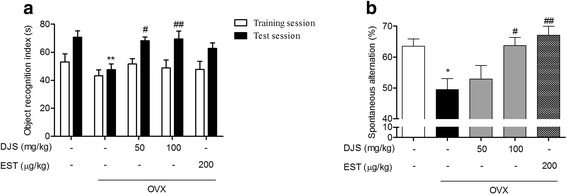
Effects of DJS on OVX-induced cognitive decline. OVX-induced cognitive decline was determined by novel object recognition test and Y-maze test. Times spent on novel and familiar objects were measured on the novel object recognition test (**a**). Spontaneous alternation behavior and the number of arm entries wre measured on the Y-maze test (**b**). *p < 0.05 and **p < 0.01 compared with the sham group, #*p* < 0.05 and ##p < 0.01 compared with the OVX group

To examine the effects of DJS-stimulated hippocampal estrogen on memory function, we performed NORT and Y-maze test, which measured cognitive function and spatial working memory of rodents [38]. In NORT, the time spent exploring a novel object in the OVX group was shorter than that of the sham group (Fig. 2A). Both the DJS treatments at 50 and 100 mg/kg/day led to increased time of exploring the novel object during the test session compared to that of the OVX group. In the Y-maze test, the percentage of spontaneous alternation in the OVX group was significantly lower than that of the sham group (Fig. 2B). The DJS-treated group with 100 mg/kg/day significantly showed a higher percentage of alternation than that of the OVX group. Together, we show that the DJS attenuated memory impairment induced by estrogen deprivation in mice.

### DJS induces CREB phosphorylation in OVX mice

**Fig. 3:**
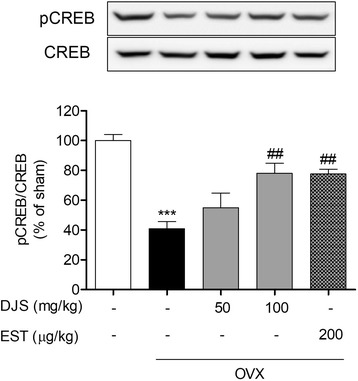
Effects of DJS on activation of CREB signaling in the mouse hippocampus. Expression of pCREB and CREB was assessed by Western blot analysis using pCREB and CREB antibodies. ***p < 0.001 compared with the sham group, ##*p* < 0.01 compared with the OVX group

Hippocampal estrogen facilitates phosphorylation of CREB, which is a nuclear protein that is required for memory formation and consolidation [39, 40]. We investigated whether DJS activates CREB expression under estrogen deprivation status. The ratio of pCREB/CREB in the OVX group was significantly reduced compared with those in the sham group; however, the DJS at 100 mg/kg/day significantly restored this ratio with similar to EST-treated group (Fig. 3).

### DJS restores OVX-induced synaptic loss in the mouse hippocampus

**Fig. 4:**
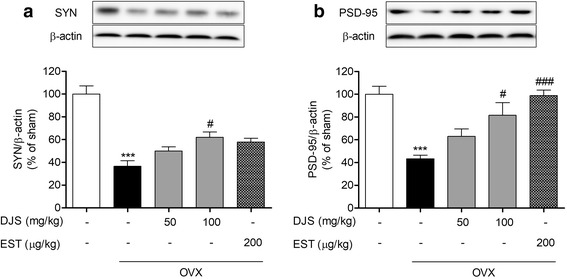
Effects of DJS on OVX-induced synaptic loss in the mouse hippocampus. Expression of synaptophysin (SYN) and PSD-95 was assessed by Western blot analysis using SYN (**a**) and PSD-95 (**b**) antibodies. ***p < 0.001 compared with the sham group, #p < 0.05 and ###p < 0.001 compared with the OVX group

It has been reported that estrogen modulates spine synapse formation in the hippocampal pyramidal neurons, which are regions for a key role in learning and memory [41]. To investigate the ameliorating effects of DJS on synaptic damage induced by OVX, we measured the expression levels of presynaptic (SYN) and postsynaptic (PSD-95) in the mouse hippocampus. We found that treatment of DJS at 100 mg/kg also significantly elevated the expression levels of SYN and PSD-95 compared to those in the OVX group (Fig. 4).

## Discussion

In the present study, we showed that DJS attenuated memory deficits and facilitated CREB and synaptic activation via stimulation of hippocampal estrogen synthesis in OVX mice. Growing evidence indicates that estrogen plays a key role in memory function by enhancing the expression of synaptic proteins and LTP-mediated synaptic plasticity in the hippocampus [14, 23, 42]. It was first demonstrated that estrogen can be synthesized de novo in the hippocampus by Fester et al. and hippocampus-derived estrogen is more closely linked to synaptic plasticity than that derived from the gonads [37, 43]. It has been reported that DJS increases estrogen levels in the blood of senescence accelerated mouse-prone 8 mice and OVX rats [31, 44]. The dose and duration of DJS administration in these studies were much higher and longer than in our study [31, 44]. Actually, the treatment of DJS in our study showed no change in serum estrogen levels (Additional file 2). Thus, we assume that the estrogen levels in the blood may depend on the dose and duration of DJS treatment.

To investigate the mechanism underlying DJS amelioration of OVX-induced memory deficits in mice, we analyzed the expression levels of pCREB, SYN, and PSD-95 in the hippocampus. The phosphorylation of CREB stimulates the synthesis of proteins such as SYN and PSD-95, which are related to synapse formation, resulting in memory enhancement [45–47]. SYN is an essential membrane glycoprotein that exists in presynaptic vesicles of neurons [48]. SYN as presynaptic marker is closely connected with cognitive function, and altering of this marker is important for synaptic connectivity and plasticity [49]. It has been reported that change of estrogen levels can affect SYN expression [41]. We confirmed that DJS could induce SYN expression via the increase of hippocampal estrogen levels, resulting amelioration of memory impairment in OVX mice. These results suggest that SYN works as a mediator of ameliorating memory deficits after DJS treatment in OVX mice.

Astrocytes, which are the most abundant glial cells in the mammalian brain, involve in the metabolic control of estrogen and has the potential to mediate neuroprotective actions of estrogen [50–52]. Thus, astrocyte activation is closely associated with estrogen signaling in brain. We found that the number of glial fibrillary acidic protein (GFAP) as an astrocyte marker-positive cells in the OVX group was significantly reduced compared with those in the sham group, however, the DJS treatment significantly restored the number with similar to EST-treated group (Additional file 3). These results suggest that DJS treatment has the possibility that induce estrogen metabolism in astrocytes as well as in neurons.

In this study, we demonstrated that DJS significantly ameliorated the memory deficits in OVX mice, by increasing the memory-related factors such as CREB and synaptic proteins via inducing the hippocampal estrogen synthesis. Previous studies have shown that hippocampal estrogen increases memory function via regulation of presynaptic and postsynaptic protein expression [23, 53]. Kato et al. reported that all sex steroids including estrogen are much higher in the hippocampus than in the blood [54]. Several studies have also shown that hippocampus-derived estrogen has a key role in synaptic plasticity, increasing spine synapse and expression of synaptic proteins, thereby improving memory function [14, 18, 55]. Given the evidence on the role of hippocampal estrogen, it is important that hippocampal estrogen shows the higher contribution to memory function than peripheral estrogen, and DJS can upregulate hippocampal estrogen.

## Conclusion

In conclusion, our results demonstrated that DJS ameliorated OVX-induced memory dysfunction via inducing hippocampal estrogen synthesis, elevating the expression of CREB and synaptic proteins. We also suggest that DJS could be a potential candidate for preventing and treating memory decline in postmenopausal women.

## Additional files

Additional file 1: Experimental design for surgery, drug administration and behavioral test (PDF 43 kb)

Additional file 2: DJS did not induce serum estrogen synthesis in OVX mice (PDF 132 kb)

Additional file 3: Effects of DJS on OVX-induced decrease of astrocyte activation in the mouse hippocampus (PDF 90 kb)

Additional file 4: Additional information (PDF 32 kb)

## Abbreviations

CREB: cAMP response element-binding protein
DJS: Dangguijakyak-san
ELISA: Enzyme-linked immunosorbent assay
ERK: Extracellular-signal-regulated kinase
EST: 17β-estradiol
LTP: Long-term potentiation
NM: Neurobasal media
NORT: Novel object recognition test
OVX: Ovariectomized
P/S: Penicillin-streptomycin
pCREB: Phosphorylated CREB
PSD-95: Postsynaptic density protein-95
SYN: Synaptophysin
