# Supplementary material for: Dangguijakyak-san ameliorates memory deficits in ovariectomized mice by upregulating hippocampal estrogen synthesis
Source: BMC Complement Altern Med. 2017 Nov 25;17:501. doi: 10.1186/s12906-017-2015-6 (PMC5702078; doi:10.1186/s12906-017-2015-6)
Supplement: Supplementary file 1 — Experimental design for surgery, drug administration and behavioral test (PDF 43 kb) [file 12906_2017_2015_MOESM1_ESM.pdf]

Additional file 1

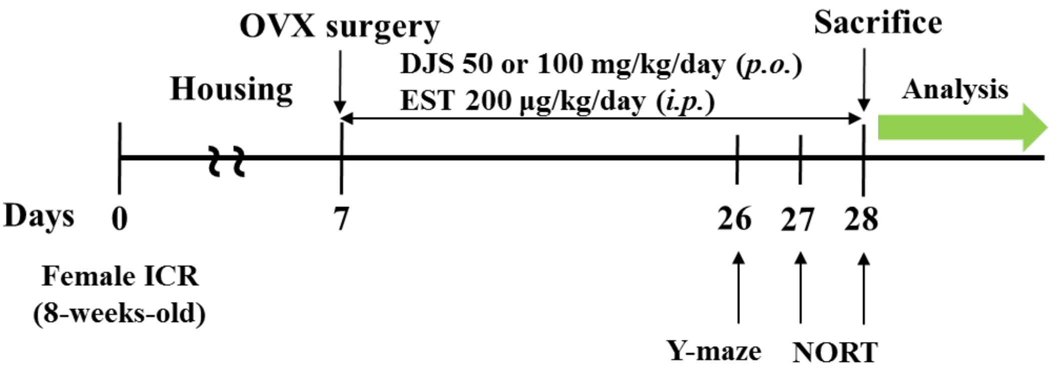

Additional file 1. Experimental design for surgery, drug administration and behavioral test. OVX: ovariectomized; DJS: dangguijakyak-san; EST: 17β-estradiol; NORT: novel object recognition test.
