# Supplementary material for: Dangguijakyak-san ameliorates memory deficits in ovariectomized mice by upregulating hippocampal estrogen synthesis
Source: BMC Complement Altern Med. 2017 Nov 25;17:501. doi: 10.1186/s12906-017-2015-6 (PMC5702078; doi:10.1186/s12906-017-2015-6)
Supplement: Supplementary file 2 — DJS did not induce serum estrogen synthesis in OVX mice (PDF 132 kb) [file 12906_2017_2015_MOESM2_ESM.pdf]

Additional file 2

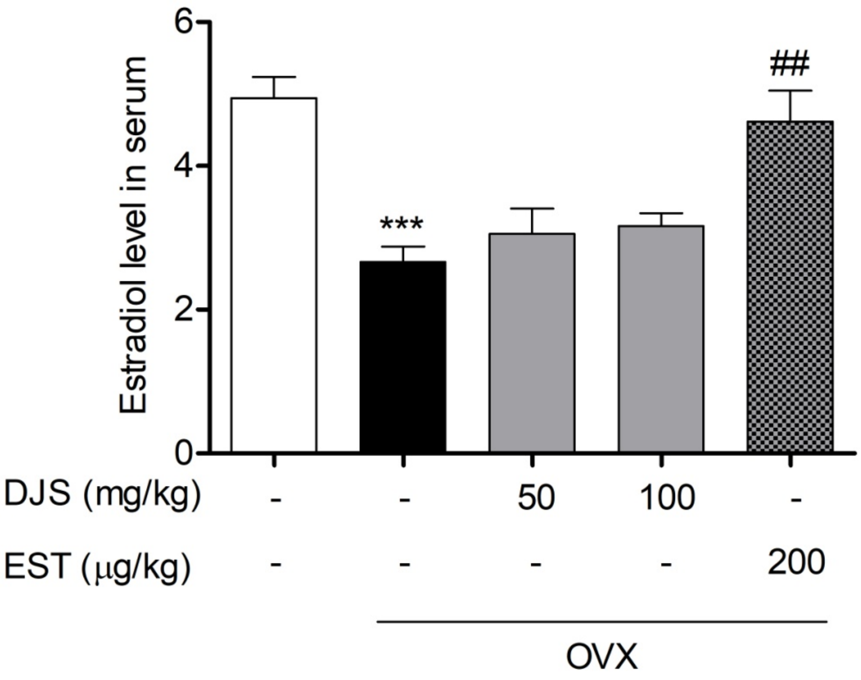

Additional file 2. DJS did not induce serum estrogen synthesis in OVX mice. Female mice were administrated with DJS at the concentration of 50 and 100 mg/kg/day and 17β-estradiol at 200 μg/kg/day for 21 days after OVX surgery. Then, 17β-estradiol levels in the seum were measured by enzyme-linked immunosorbent assay. Values are means ± standard error. ***p < 0.001 compared with the sham group, ##p < 0.01 compared with the OVX group.
