# Supplementary material for: Dangguijakyak-san ameliorates memory deficits in ovariectomized mice by upregulating hippocampal estrogen synthesis
Source: BMC Complement Altern Med. 2017 Nov 25;17:501. doi: 10.1186/s12906-017-2015-6 (PMC5702078; doi:10.1186/s12906-017-2015-6)
Supplement: Supplementary file 3 — Effects of DJS on OVX-induced decrease of astrocyte activation in the mouse hippocampus (PDF 90 kb) [file 12906_2017_2015_MOESM3_ESM.pdf]

Additional file 3

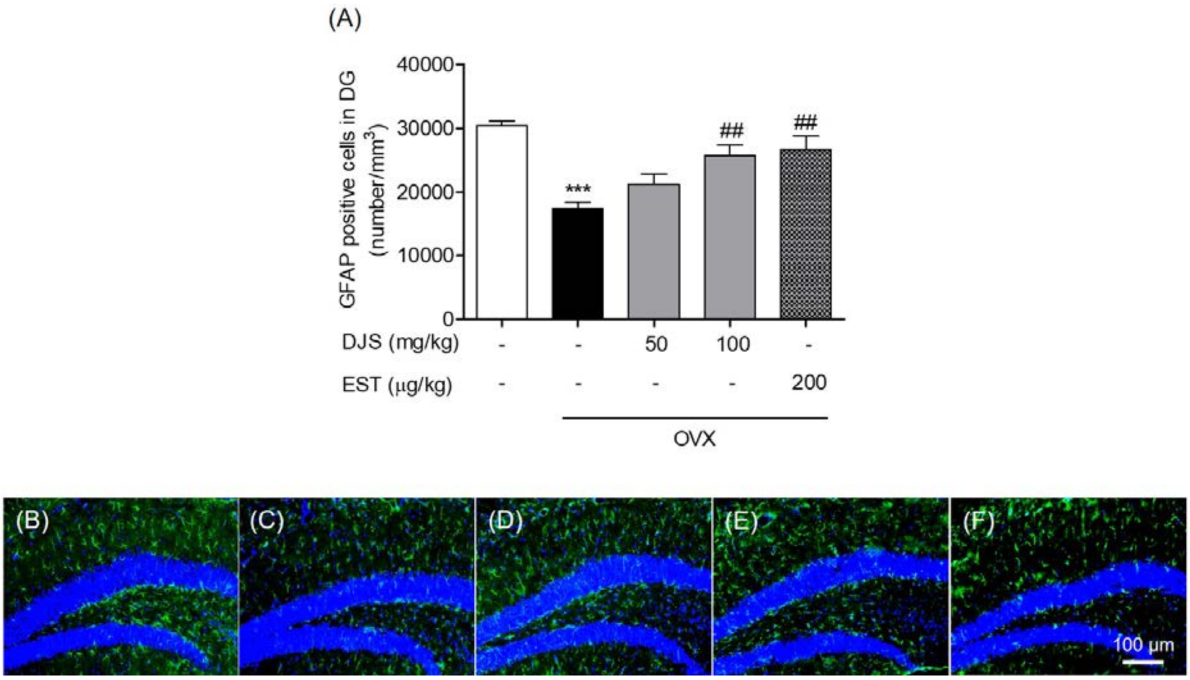

Additional file 3. Effects of DJS on OVX-induced decrease of astrocyte activation in the mouse hippocampus. Astrocyte activation was assessed by GFAP immunofluorescence staining (A). Each representative image was shown as follows; sham-operated (B), OVX (C), OVX+DJS 50 mg/kg (D), OVX+DJS 100 mg/kg (E), and OVX+EST 200 μg/kg (F). Scale bar = 100 μm. ***p < 0.001 compared with the sham group, ##p < 0.01 compared with the OVX group.
