# Supplementary material for: Dangguijakyak-san ameliorates memory deficits in ovariectomized mice by upregulating hippocampal estrogen synthesis
Source: BMC Complement Altern Med. 2017 Nov 25;17:501. doi: 10.1186/s12906-017-2015-6 (PMC5702078; doi:10.1186/s12906-017-2015-6)
Supplement: Supplementary file 4 — Additional information (PDF 32 kb) [file 12906_2017_2015_MOESM4_ESM.pdf]

Additional information

Immunofluorescence

For the immunofluorescence study of GFAP, every fifth brain section was taken from the region between bregma -1.70 mm and bregma -2.30 mm; this was performed for each brain [1]. Brain sections were briefly rinsed in PBS buffer. Next, the sections were incubated with a goat anti-GFAP antibody (1:1000 dilution) overnight at 4 °C in the presence of 1% triton X-100 and 1% BSA. After rinsing in PBS buffer, the sections were then incubated with a Alexa 594 chicken anti-goat IgG (1:500 dilution) for 70 min at room temperature. Then, the sections were incubated with 4′,6-diamidine-2′-phenylindole dihydrochloride (DAPI) staining solution (1:5000 dilution) for 20 min at room temperature. Stained sections were mounted on gelatin-coated slides and coverslipped using fluorescent mounting medium. The images were photographed using a fluorescence microscope (BX51, Olympus, Japan). The number of GFAP-positive cells in the DG of mouse hippocampus was quantified according to stereological counting and they were analyzed with Image J software [2].

References

1. Franklin K, Paxinos G: The Mouse Brain in Stereotaxic Coordinates: Academic Press; 1997.
2. Mura A, Murphy CA, Feldon J, Jongen-Relo AL: The use of stereological counting methods to assess immediate early gene immunoreactivity. *Brain Res* 2004, 1009(1-2):120-128.
